# RANKL Is Involved in Runx2-Triggered Hepatic Infiltration of Macrophages in Mice with NAFLD Induced by a High-Fat Diet

**DOI:** 10.1155/2020/6953421

**Published:** 2020-05-25

**Authors:** Li Zhong, Jianghan Yuan, Lu Huang, Shan Li, Liang Deng

**Affiliations:** ^1^Department of Gastroenterology, The First Affiliated Hospital of Chongqing Medical University, Chongqing, China; ^2^Department of Gastroenterology and Hepatology, The Second Affiliated Hospital of Chongqing Medical University, Chongqing, China; ^3^Department of Critical Care Medicine, The First Affiliated Hospital of Chongqing Medical University, Chongqing, China; ^4^Chongqing Key Laboratory of Child Infection and Immunity, China; ^5^Department of Pediatric Research Institute, China; ^6^Ministry of Education Key Laboratory of Child Development and Disorders, Children's Hospital of Chongqing Medical University, Chongqing, China

## Abstract

**Background:**

Receptor activator of nuclear factor-*κ*B (NF-*κ*B) ligand (RANKL) is significant in the activation of inflammation. Runt-related transcription factor 2 (Runx2) promotes the hepatic infiltration of macrophages in nonalcoholic fatty liver disease (NAFLD). We studied how RANKL affects Runx2-triggered macrophage infiltration in NAFLD.

**Method:**

30 male C57BL/6J mice at 4 weeks of age were utilized in this study, 20 mice received a high-fat diet (HFD), and 10 mice received standard rodent chow over 8 months. The histopathologic features of the liver were identified by H&E, Oil red O, and Masson staining. Runx2, RANKL, and F4/80 were analyzed by western blot, real-time PCR, and immunohistochemistry *in vivo*, respectively. Lentivirus or siRNA was utilized for transwell assay to investigate the role of RANKL in Runx2-induced macrophage migration *in vitro*.

**Results:**

Compared to controls, during NAFLD development, HFD increased Runx2 and RANKL *in vivo* in NASH (*P* < 0.01). Meanwhile, a correlation between the expression of Runx2 and RANKL (*P* < 0.05) was evident. In addition, the hepatic infiltration of macrophages was increased upon HFD feeding, and analysis showed that the macrophage infiltration was correlated with the expression of Runx2 or RANKL (*P* < 0.05). *In vitro*, we found that overexpression or deficiency of Runx2 increased or decreased the production of RANKL in mHSCs. Then, transwell assay revealed that RANKL was involved in Runx2-induced macrophage migration.

**Conclusions:**

Overall, RANKL is involved in Runx2-triggered macrophage migration during NAFLD pathogenesis, which may provide an underlying therapeutic target for NAFLD.

## 1. Background

Nonalcoholic fatty liver disease (NAFLD) presents a high burden of liver disease worldwide, incorporating various levels from simple steatosis to nonalcoholic steatohepatitis (NASH) and finally cirrhosis. NASH remains a significant trigger of chronic liver disease [1–4]. Evidence demonstrates that inflammation and hepatocytic injury play essential roles in the progression of NAFLD and that immune activation is critical in the evolution of NAFLD to NASH, particularly in the activation of macrophages [5, 6]. Generally, macrophages are hepatic sinusoid residents and predominantly nonmigratory phagocytes. Besides, they are usually recruited by several chemokines and expand rapidly during the development of NAFLD. Macrophages control the initiation and progression of NAFLD through releasing several inflammatory factors or chemokines, such as monocyte chemotactic protein 1 (MCP-1), interleukin-1*β*, and tumor necrosis factor *α* [7–10]. In mice fed HFD, the recruited macrophages promote liver steatosis and inflammation, which argues the influence of macrophage infiltration on the evolution of NAFLD [11, 12].

Runt-related transcription factor 2 (Runx2) is the main regulator in osteoblast differentiation. Runx2 also acts to diversify functions in various other tissues, Runx2 regulates vascular calcification in vessels, and it might constitute a major driving force in tumor progression and aggressiveness [13–17]. According to our previous studies, Runx2 is cell-specific elevated in activated murine hepatic stellate cells (mHSCs) in NAFLD mice and induces macrophage migration in vitro, partially through upregulating MCP-1 [18]. However, the silencing of MCP-1 did not completely block the migration of macrophages induced by Runx2, which indicated that there might be other factors involved in this mechanism.

Receptor activator of nuclear factor-*κ*B (NF-*κ*B) ligand (RANKL), or TNFSF11, is a tumor necrosis factor which is a key regulator for osteoclast formation [19]. RANKL functions are usually focused on bone homeostasis, immune tolerance, and cancer, but its effects are still undiscovered in NAFLD. A recent study has demonstrated that RANKL signaling contributes to innate immune activation in primary biliary cholangitis patients [20]. In one case, the administration of the anti-RANKL antibody during osteoporosis treatment in a female patient with growth hormone deficiency resulted in the amelioration of hepatitis [21]. RANKL is also found to be related to metabolic disease, as blocking RANKL recovered hepatic insulin resistance in mice [22]. As hepatic insulin resistance is significant during the progression of NAFLD, we hypothesized that RANKL might contribute to the development of NAFLD. Interestingly, RANKL can be regulated by Runx2 in smooth muscle cells and is involved in promoting macrophage migration during vascular calcification [23]. Therefore, we contemplated that in NAFLD, RANKL might be related to the Runx2-induced hepatic infiltration of macrophages.

Based on the disease stage-dependent manner, we revealed that HFD visibly increased the hepatic permeation of macrophages, Runx2, and RANKL *in vivo*, especially in the NASH stage. Meanwhile, the analysis showed there was a correlation among Runx2 expression, RANKL production, and hepatic macrophage infiltration. In addition, we found that Runx2 promoted the migration of macrophages partially by upregulating RANKL *in vitro*. These outcomes, altogether, demonstrate that RANKL is involved in Runx2-induced hepatic infiltration of macrophages in NAFLD, which reveals an underlying therapeutic target for NAFLD.

## 2. Material and Methods

### 2.1. Animals and Diets

The expression of Runx2 and the hepatic infiltration of macrophages were studied previously in a time-dependent manner during NAFLD development. However, due to the individual variation of mice, hepatic histological features might present different stages of NAFLD even after a consistent treatment of HFD. This could influence the analysis of Runx2 expression or the hepatic macrophage recruitment in each stage of NAFLD. Therefore, in this study, we generated mice with NAFLD by feeding with HFD and then divided the mice into control, simple steatosis (NAFLD), and NASH groups, according to the hepatic histological features. A total of thirty 4-week-old male C57BL/6J mice were included from the Experimental Animal Center of Chongqing Medical University. These mice were divided at random into a high-fat diet group (HFD, *n* = 20) and a standard rodent chow group (control, *n* = 10), and they were provided free access to feed and water. The diet was maintained for eight months, and then, the mice were sacrificed for testing. The investigators maintained the standard rodent chow and HFD (TROPHIC Animal Feed High-Tech Co. Ltd., China) in 4°C conditions and renewed the chow every other day. The main components (g/kg) of the two diets were presented in a previous study [18]. All experiments followed the guidelines on the humane treatment of animals established by Chongqing Medical University.

### 2.2. Enzyme-Linked Immunosorbent Assay

The blood samples or culture supernatant liquid of mHSC from the different groups were collected and separated by centrifugation (1000 × g; 10 minutes). The RANKL concentrations were measured by Mouse RANKL ELISA Kit (Catalog #EK0843, Boster Biological Technology, Wuhan, China).

### 2.3. Tissue Preparation and Histological Examination

The liver samples were divided into pieces and fixed in 4% paraformaldehyde for Hematoxylin-Eosin (H&E), Masson's trichrome staining, and immunohistochemical staining. Frozen sections were applied for Oil red O staining. H&E, Oil red O, and Masson's trichrome staining were processed routinely with commercial kits (Solarbio, China).

The histopathologic features observed were used to stage the evolution of NAFLD using the NAFLD activity score (NAS) under a microscope with ×200 magnification. The standard criteria for determining the stage for NASH were based on hepatic steatosis, hepatocellular injury, and inflammation [24]. A NAS score < 3 was not deemed NASH. Meanwhile, a NAS score ≥ 5 was considered NASH.

### 2.4. Immunohistochemistry

For antigen retrieval, sections were dewaxed, rehydrated, and warmed with a citrate buffer. Next, to block endogenous peroxidase, they were incubated with peroxide. Investigators stained the sections with anti-F4/80 antibody (1/300 dilution; MCA497R, Serotec, Raleigh, NC, USA) overnight at 4°C after blocking with goat serum. The next day, the immunoperoxidase technique, using match application, with Envision kits (Boster Biological Technology, China) was performed. These samples were then stained with diaminobenzidine and hematoxylin. The positive stained cells of F4/80 of sections were counted using ×200 magnification.

### 2.5. Western Blot

The liver tissues of mice in each group or mHSCs were homogenized on ice in a lysis buffer, which contained 20 mM Tris-HCl, pH 7.4, 1% SDS, and 1% of protease inhibitor. The investigators resolved the protein extracts on SDS-PAGE. These samples were then transferred onto polyvinylidene difluoride membranes where they underwent incubation with primary antibodies against Runx2 (1 : 1000 dilution; ab76956, Abcam, Cambridge, UK), F4/80 (1 : 600 dilution), RANKL (1 : 600 dilutions; BA1323, Boster Biological Technology, Wuhan, China), and GAPDH (1 : 1000 dilutions; BM3876, Boster Biological Technology, Wuhan, China) overnight at 4°C. The investigators applied the horseradish peroxidase-coupled secondary antibodies (Boster Biological Technology, China) at 1 : 5000 dilutions at room temperature for 90 minutes. The proteins were visualized using a chemiluminescence kit (Millipore, Billerica, MA, USA). Protein expression was quantified by using the Molecular analyst software (Bio-Rad, Marne-la-Coquette, France).

### 2.6. Quantitative Real-Time Polymerase Chain Reaction (PCR)

The investigators extracted the total RNA from liver tissues or mHSCs with the RNeasy Mini Kit (Qiagen, Valencia, CA, USA). These underwent reverse transcription into their complementary DNA using the PrimeScript® RT reagent Kit (RR047A, Takara Bio, Japan). Quantitative real-time PCR was performed using SYBR Premix Ex Taq™ Kit (TaKaRa, Japan) according to the manufacturer's procedure. Specific oligonucleotide primers were used as follows: 5′-CAGGAAGACTGCAAGAAGGCTCTGG-3′ (forward) and 5′-ACACGGTGTCACTGCGCTGAAGA-3′ (reverse) for Runx2, 5′-TGTACTTTCGAGCGCAGATG-3′ (forward) and 5′-ACATCCAACCATGAGCCTTC-3′ (reverse) for RANKL, and 5′-AGTACGATGTGGGGCTTTTG-3′ (forward) and 5′-CCCCATCTGTACATCCCACT′ (reverse) for F4/80. The reaction conditions were as follows: 95°C for one minute; 39 cycles of 95°C for thirty seconds, 60°C for thirty seconds, and 72°C for thirty seconds; and 72°C for two minutes.

### 2.7. Cell Culture and Transfection

In a humidified atmosphere of 5% CO_2_, the investigators cultured murine hepatic stellate cell line (mHSC) and macrophage line Raw264.7 in DMEM with 10% FBS and 1% penicillin/streptomycin. The shRNA lentivirus (Lenti-shRunx2) targeting the mice Runx2 gene was then constructed by Genechem (Shanghai, China), and the shRNA sequence was 5′-GCCCAGGCGTATTTCAGATGA-3′. The lentiviral expression vector (Lenti-Runx2) to induce overexpression of Runx2 was assembled by OmicsLink™ (GeneCopoeia, Guangzhou, China).

The investigators seeded mHSCs in 6-well plates, where they infected the samples with control lentivirus (Lenti-ctrl), Lenti-shRunx2, or Lenti-Runx2 using the Polybrene (GeneCopoeia, Guangzhou, China) system following the manufacturer's procedure. Infected cells were chosen using 3 *μ*g/ml puromycin for later studies.

To detect soluble RANKL, distinct samples of mHSCs were cultured using a density of 4 × 10^5^ cells/well in 6-well plates for twenty-four hours. This was then replaced by a serum-free medium. The supernatants were gathered at forty-eight hours after incubation. ELISA for RANKL was then performed.

### 2.8. Cell Migration Assay

To analyze the migration of Runx2 and RANKL in mHSCs on the accumulation of macrophages, the investigators completed transwell assays (8 *μ*m pore size; Corning Inc.). The investigators harvested mHSCs transfected with Lenti-ctrl, Lenti-shRunx2, and Lenti-Runx2 by trypsinization. These were seeded in 24-well plates. After twenty-four hours, these were transfected with RANKL siRNA or controls as per the manufacturer's guidelines. The siRNA vector targeting mouse RANKL or MCP-1 was created by Genepharma (Shanghai, China). The target sequences for RANKL siRNA are 5′-CCAUCAAUGCUGCCAGCAUTT-3′ and 5′-AUGCUGGCAGCAUUGAUGGTT-3′, and the scramble sequences are 5′-UUCUCCGAACGUGUCACGUTT-3′ and 5′-ACGUGACACGUUCGGAGAATT-3′. The target sequences for MCP-1 were described in a previous study [18]. After 8 hours, the siRNA transfection mixture was replaced with fresh medium and 1 × 10^5^ Raw264.7 cells per well were seeded on top of the filter and cultured for 36 hours. Then, the macrophages above the filter were erased, and the macrophages below the filter were stained with crystal blue. The average positive macrophage cell amount over 3 visual samples for each transwell was taken as the positive cell number in the group.

### 2.9. Statistical Analysis

In this study, the data were analyzed using SPSS 19.0 software and presented as the mean ± SD. The significance of differences between groups was determined by one-way analysis of variance. *P* < 0.05 was considered statistically significant.

## 3. Results

### 3.1. Histopathologic Features

**Figure 1:**
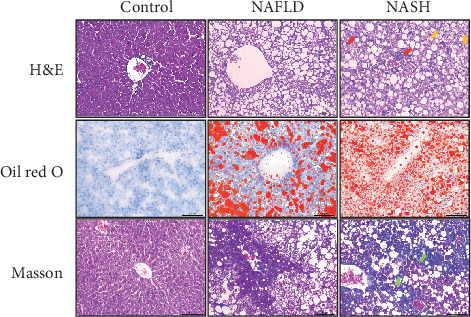
Histopathologic features of C57BL/6 mice on HFD or control diet over 8 months. H&E staining, Oil red O staining, and Masson's trichrome staining were performed. Representative images were amplified 200 times. Hepatocyte ballooning was pointed out by yellow arrows, lobular inflammation was marked by red arrows, and fibrosis was labelled by green arrows. *n* = 10 for each group.

The histopathologic features were identified by H&E, Oil red O, and Masson's trichrome staining and appraised using the NAFLD activity score (NAS). The mice in the HFD group were divided into two categories, according to NAS: NAFLD (simple steatosis) and NASH. As shown in Figure 1, all mice provided HFD presented the pathological features of obvious macrovesicular steatosis after 8 months, while half evolved into NASH (NAS ≥ 5) (50%) with pathological features of not only steatosis but also prominent hepatocyte ballooning and lobular inflammation or fibrosis.

### 3.2. Both RANKL and Runx2 Increased during the Progression of NAFLD

**Figure 2:**
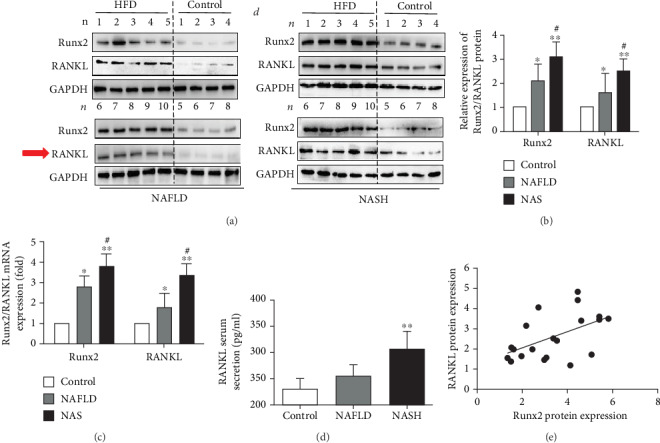
Both RANKL and Runx2 increased during NAFLD development. (a) RANKL and Runx2 protein expressions in the liver were identified by western blot. (b) Statistical analysis of the protein expression of RANKL and Runx2. (c) Relative mRNA expressions of RANKL and Runx2 were detected by Real-Time PCR. (d) Serum productions of RANKL were measured by ELISA. (e) The correlation between Runx2 and RANKL protein expression in the HFD group. 8 ≤ *n* ≤ 10. ^∗^*P* < 0.05 vs. controls, ^∗∗^*P* < 0.01 vs. controls, and ^#^*P* > 0.05 vs. NAFLD.

Runx2 and RANKL expressions in vivo were identified by western blot. An HFD elevated Runx2 and RANKL in both mRNA and protein levels with statistical differences (*P* < 0.05) (Figures 2(a) and 2(c)). Interestingly, the expression of Runx2 and RANKL was higher in the NASH group compared with NAFLD, but without statistical differences (*P* > 0.05). (Figures 2(b) and 2(c)). Serum productions of RANKL were similar to the tendency of mRNA and protein expression, but the elevation in NAFLD mice was not statistically significant compared to controls (Figure 2(d)). Besides, the correlation between Runx2 and RANKL expression was analyzed in the HFD group, and the results showed RANKL was upregulated and accompanied Runx2 (*P* < 0.05) (Figure 2(e)). Both Runx2 and RANKL expressions increased along with the progression of NAFLD, which were moderately elevated in the early stage of NAFLD and obviously aggravated in NASH.

### 3.3. Hepatic Infiltration of Macrophages Accompanies the Elevation of Runx2 and RANKL during NAFLD Development

**Figure 3:**
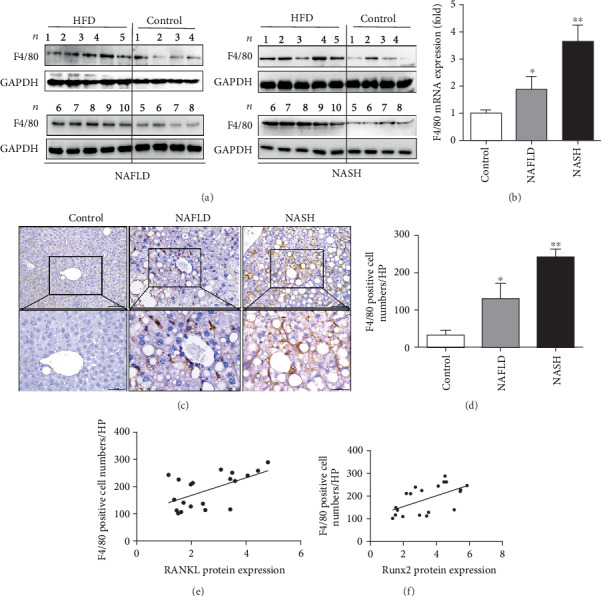
Hepatic infiltration of macrophages accompanies a rise in Runx2 and RANKL during NAFLD development. (a) Western blot detected F4/80 protein expression in liver tissues. (b) Real-time PCR detecting the mRNA expression of F4/80 in liver tissues. (c) Liver sections immunohistochemical staining for F4/80; representative images were amplified 200 and 400 times. (d) Quantification for F4/80^+^ cells presented as a total number of positive cells/field (×200). (e) The correlation between RANKL protein expression and F4/80-positive cells in each sample in the HFD group. (f) The correlation between Runx2 protein expression and F4/80+ cells in each sample in the HFD group. 8 ≤ *n* ≤ 10. ^∗^*P* < 0.05 vs. controls; ^∗∗^*P* < 0.01 vs. controls.

In the present study, the infiltration of macrophages during the progression of NAFLD was analyzed by detecting F4/80, a precise indicator for murine macrophages [25]. First, we measured the F4/80 expression among the different categories of liver tissues. As shown in Figures 3(a) and 3(b), when compared to the controls, F4/80 increased in the NAFLD and was significantly elevated in the NASH group (3.7-fold increase vs. controls) (*P* < 0.01). Furthermore, we analyzed macrophage infiltration through immunohistochemistry. The results showed that F4/80-positive cells were rarely observed in the controls but increased in frequency in the NAFLD group, then were markedly provoked in the NASH group, similar to the tendency of F4/80 mRNA and protein expression (Figures 3(c) and 3(d)). As NAFLD developed, the amount of F4/80-positive cells increased in the liver, which followed the trends of Runx2 and RANKL expression. Thus, we analyzed the correlations of the expression of Runx2 or RANKL with the F4/80-positive cells. The results suggested that the expression of Runx2 or RANKL significantly correlated with the F4/80-positive cells, which indicated hepatic infiltration of macrophages accompanied the elevation of Runx2 and RANKL during NAFLD development (*P* < 0.05) (Figures 3(e) and 3(f)).

### 3.4. RANKL Involved in Runx2-Induced Macrophage Recruitment *In Vitro*

**Figure 4:**
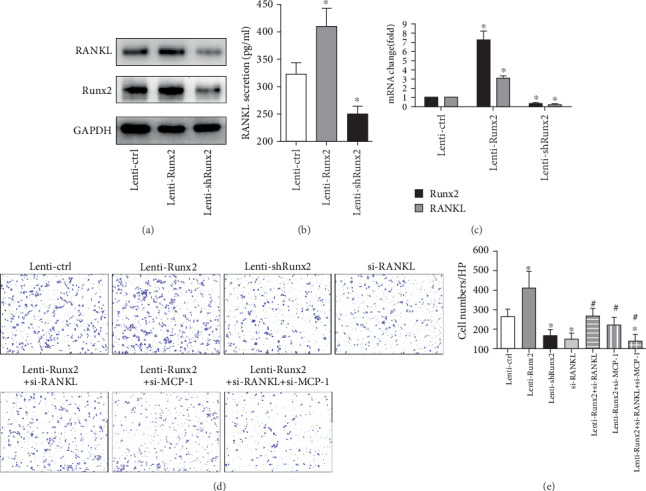
RANKL involved in Runx2-induced macrophage recruitment in vitro. mHSCs infected with lentivirus carrying control scrambled shRNA (Lenti-ctrl), lentivirus overexpressed vector (Lenti-Runx2), or shRNA specific for Runx2 (Lenti-shRunx2) for regulation of Runx2. siRNA silenced RANKL or MCP-1 in mHSCs. (a) Western blot of mHSCs infected with lentivirus to detect the protein expression of Runx2 and RANKL. (b) ELISA results on mHSCs infected with lentivirus after forty-eight hours, to detect the concentration of soluble RANKL. (c) The mRNA expressions of Runx2 and RANKL were observed via real-time PCR in mHSCs transfected with Lenti-ctrl, Lenti-Runx2, or Lenti-shRunx2. (d) mHSCs treated with lentivirus for Runx2 or siRNA for RANKL or MCP-1 and cocultured with macrophages to perform transwell assay; images were amplified 200x. (e) Quantification for migrated macrophages per field (×200). Results from 3 independent experiments are presented. ^∗^*P* < 0.05 vs. controls and ^#^*P* < 0.05 vs. controls.

The mRNA expression of RANKL in primary HSC was increased while HSC was activated *in vitro* (data was not shown). This suggested RANKL might be one of the factors secreted from activated HSC. To determine whether RANKL was involved in Runx2-induced hepatic infiltration of macrophages, overexpression and shRNA lentivirus of Runx2 were utilized in vitro. The transfection efficiency of the lentivirus system in HSCs was evaluated by luciferase activity. As expected, increasing Runx2 decidedly upregulated RANKL protein and mRNA expression, while the knockdown of Runx2 significantly diminished the expression of RANKL in mHSC (*P* < 0.05) (Figures 4(a) and 4(c)). Additionally, Runx2 also influenced RANKL secretion in mHSCs (*P* < 0.05) (Figure 4(b)). By using the transwell coculture system of mHSCs and macrophages, we found that increased Runx2 stimulated macrophage migration, while a deficiency of Runx2 or RANKL decreased macrophage migration. More importantly, the silencing of RANKL attenuated Runx2-induced migration of macrophages in mHSCs (Figures 4(d) and 4(e)). As earlier data showed that MCP-1 was involved in Runx2-induced macrophage migration, we separately silenced MCP-1 or RANKL in Runx2-overexpressed mHSCs, and both of them could attenuate macrophage migration. Also, deficiency of MCP-1 and RANKL in Runx2-overexpressed mHSCs further decreased macrophage migration (Figures 4(d) and 4(e)). This outcome indicated that Runx2 regulated RANKL expression, and RANKL was involved in Runx2-induced macrophage migration *in vitro*.

## 4. Discussion

As there are robust links between NAFLD and obesity [26], our experiment induced NAFLD via an HFD in mice to evaluate NAFLD progression. Based on our previous research, mice eventually induced NASH after 8 months of high-fat feeding. To further explore the relationship between the HFD and NAFLD progression, the mice were sacrificed after 8-month HFD in the present study. All the mice fed the HFD were found to have typical steatosis, and half of them developed to NASH stage with obvious inflammation, hepatocyte ballooning, or even fibrosis. Recent studies have hypothesized that hepatic macrophages are the trigger of hepatic fat accumulation and are considered to encourage NAFLD progression to NASH [9, 27]. At the beginning of NAFLD, hepatic macrophages increase precipitously, due to chemokines released by Kupffer cells, hepatocytes, and activated HSCs. Removal of these macrophages attenuates the inflammation and prevents the development of NAFLD [7–9, 28–30]. In our study, hepatic macrophage infiltration was detected by F4/80 for all macrophages in the liver. We found that macrophage infiltration initiated at NAFLD and was significantly provoked at NASH. This reveals an infiltration during the NAFLD pathogenesis. As we know, macrophage migration requires chemokines as we tried to discover which factor contributes to recruiting macrophages in NAFLD.

In previous studies, we demonstrated that Runx2 induced hepatic infiltration of macrophages via the upregulation of MCP-1 and that silencing MCP-1 expression did not completely block Runx2-recruited macrophages *in vitro*, so there must be other factors involved in this mechanism [18]. It has been confirmed that RANKL serves a significant role in recruiting bone marrow macrophages in certain metabolic diseases, like atherosclerosis and diabetes mellitus [23, 31]. Also, RANKL is considered to contribute to the mechanism of hepatitis as the blocking RANKL signaling has been even shown to ameliorate hepatitis [20, 21]. However, the function of RANKL in NAFLD and whether it is involved in the underlying mechanism of Runx2-induced hepatic macrophage infiltration remained unknown. To elucidate this hypothetical mechanism, the expressions of Runx2, RANKL, and F4/80 were examined *in vivo*. In this study, the productions of Runx2, RANKL, and F4/80 were elevated at NAFLD and significantly provoked at NASH, while there were high correlations between their changes that indicated RANKL might be associated with the Runx2-induced macrophage infiltration during NAFLD development.

Both RANKL and Runx2 are recognized as important regulators in bone formation, and Runx2 can regulate RANKL expression by directly affecting its promoter [23, 32, 33]. As previously described, Runx2 is scarcely expressed in hepatocytes but abundantly elevated in activated HSCs [18]. Thus, we chose mHSCs to confirm the conjecture, and the results showed that Runx2 regulated the production of RANKL in mHSCs, while silencing of RANKL attenuated Runx2-induced migration of macrophages *in vitro*. Expectedly, a deficiency of both RANKL and MCP-1 could further decrease but not entirely block the migration of macrophages compared to separately silencing *in vitro*. In consequence, it indicated that both of these factors were involved in Runx2-induced macrophage migration in HSC.

## 5. Conclusions

This study has elucidated that Runx2, RANKL, and macrophage infiltration are consistently increased during the progression of NAFLD, and RANKL is involved in Runx2-induced macrophage migration *in vitro*. These findings may provide an underlying therapeutic target for NAFLD.

## Data Availability

The data used to support the findings of this study are available from the corresponding author upon request.
